# The Dispersion State of Tangled Multi-Walled Carbon Nanotubes Affects Their Cytotoxicity

**DOI:** 10.3390/nano6110219

**Published:** 2016-11-19

**Authors:** Chika Kuroda, Hisao Haniu, Kumiko Ajima, Manabu Tanaka, Atsushi Sobajima, Haruka Ishida, Tamotsu Tsukahara, Yoshikazu Matsuda, Kaoru Aoki, Hiroyuki Kato, Naoto Saito

**Affiliations:** 1 Institute for Biomedical Sciences, Interdisciplinary Cluster for Cutting Edge Research, Shinshu University, 3-1-1 Asahi, Matsumoto, Nagano 390-8621, Japan; 15mz011b@shinshu-u.ac.jp (C.K.); ajima@shinshu-u.ac.jp (K.A.); 16bs203j@shinshu-u.ac.jp (H.I.); saitoko@shinshu-u.ac.jp (N.S.); 2 Physical and Occupational Therapy Division, Graduate School of Medicine, Shinshu University, 3-1-1 Asahi, Matsumoto, Nagano 390-8621, Japan; kin29men@shinshu-u.ac.jp; 3 Department of Orthopaedic Surgery, Shinshu University School of Medicine, 3-1-1 Asahi, Matsumoto, Nagano 390-8621, Japan; m990054e@shinshu-u.ac.jp (M.T.); soba@shinshu-u.ac.jp (A.S.); hirokato@shinshu-u.ac.jp (H.K.); 4 Biomedical Engineering Division, Graduate School of Science and Technology, Shinshu University, 3-1-1 Asahi, Matsumoto, Nagano 390-8621, Japan; 5 Department of Molecular Pharmacology and Neuroscience, Nagasaki University Graduate School of Biomedical Sciences, 1-14 Bunkyo-machi, Nagasaki 852-8521, Japan; ttamotsu@nagasaki-u.ac.jp; 6 Clinical Pharmacology Educational Center, Nihon Pharmaceutical University, 10281 Komuro, Ina-machi, Saitama 362-0806, Japan; yomatsuda@nichiyaku.ac.jp

**Keywords:** multi-walled carbon nanotube, tangle, sonicator, cytotoxicity, AlamarBlue assay

## Abstract

The medical applications of carbon nanotubes (CNTs) have garnered much attention. However, evaluating the safety of CNTs remains difficult, and no consensus has been reached. Moreover, assessing the biosafety of multi-walled CNTs (MWCNTs), which can become tangled during manufacturing, is challenging because they do not readily disperse. We studied how the dispersion state of tangled MWCNTs affects their cytotoxicity, using three sonicators. Flotube 9110 (FT9110), tangled MWCNTs, were dispersed in two dispersants (fetal bovine serum and polysorbate 80) using a new type of sonicator (PR-1) and two conventional sonicators. The size and cytotoxicity of the dispersed FT9110 were measured using the BEAS-2B human bronchial epithelial cell line. The PR-1 dispersed the FT9110 to agglomerates <200 nm in diameter; FT9110 dispersed with the PR-1 did not show cytotoxicity regardless of dispersant. The other sonicators dispersed the FT9110 to particles >1000 nm in diameter, and cytotoxicity depended on the dispersant. We found that excluding cells adhered to agglomerated FT9110 before evaluating cytotoxicity can lead to false-positive results. The PR-1 sonicator dispersed tangled FT9110 to many single fibers, which showed lower cytotoxicity than conventionally-sonicated MWCNTs. We suggest that dispersion state should be accounted for when evaluating the cytotoxicity of MWCNTs.

## 1. Introduction

Carbon nanotubes (CNTs) possess many unique characteristics that have attracted the interest of researchers in a broad range of fields. CNTs have useful electrical, thermal, and mechanical properties, and their material performance can be improved by combination with other materials [1,2,3,4]. In the medical field, extensive research is underway to develop new CNT biomaterials for use in the treatment and diagnosis of disease [5,6,7]. However, there are concerns about the effect of CNTs on human health [8,9,10]. Since nanomaterials show different physicochemical properties from conventional materials, their effects on human health remain poorly understood. In particular, multi-walled CNTs (MWCNTs) are similar to asbestos in shape and size, raising concern that MWCNTs may be carcinogenic in humans. Safety evaluations of MWCNTs typically use MWNT-7 (Mitsui, Tokyo, Japan), and many reports of in vivo and in vitro experiments have been published. MWNT-7 and other types of MWCNTs show carcinogenicity and cause cytotoxic and inflammatory responses [11,12,13,14]. However, MWCNTs vary in size and type, and produce different biological responses.

We are developing implants using MWCNTs, tangled-MWCNTs in particular. The safety of tangled-MWCNTs has also been investigated, and they show cytotoxicity but no carcinogenicity [13,15,16,17]. However, the tangled-MWCNTs that have been investigated were not well dispersed, making it difficult to determine whether the results reflect the effects of nanosized, fibrous MWCNTs or the effects of microsized, agglomerated MWCNTs. We also reported that tangled-MWCNTs, named VGCF-X, showed significant cytotoxicity in the BEAS-2B human bronchial epithelial cell line but little cytotoxicity in the MESO-1 human malignant pleural mesothelioma cell line [18]. However, at that time we were not able to effectively disperse tangled VGCF-X.

The dispersant is an important factor in dispersion of hydrophobic CNTs. In addition, different dispersants affect not only the size of CNT agglomerates but also biological responses, as we have previously reported [19]. On the other hand, it is well known that nanoparticles are rapidly coated with various proteins in biological fluids, forming a protein corona [20,21,22]. Therefore, some investigations have used bovine serum albumin (BSA), which is the most abundant protein in serum or plasma, as the dispersant for CNTs [23,24,25]. However, the abundance profile of proteins absorbed to CNTs did not match the protein profile of the serum [26]. Moreover, differences in biological responses when proteins and surfactants, such as polysorbate 80 (PS) and dipalmitoylphosphatidylcholine (DPPC), are used remain unclear.

In this study, we compared the efficacy of a new sonicator, with improved dispersion efficiency, with conventional sonicators for dispersion for whole tangled MWCNTs, using two dispersants, fetal bovine serum (FBS) and surfactant. Sonicated MWCNTs were evaluated for cytotoxicity on the BEAS-2B cell line, which was previously shown to be more sensitive than the MESO-1 cell line. The findings indicate that tangled MWCNTs can be effectively dispersed by the new sonicator and that the difference in dispersion methods affected cytotoxicity. Moreover, MWCNTs dispersed in FBS produced a different biological response from those dispersed in surfactant.

## 2. Results

### 2.1. Characterization of Dispersed FT9110

We compared a new sonicator, the PR-1 (Thinky, Tokyo, Japan), with conventional sonicators for dispersion of tangled MWCNTs. The PR-1 employs a water bath, and we, therefore, used an US-1R sonicator (As one, Tokyo, Japan), which is similar, for comparison. Since the output power of the PR-1 is 140 W, we also compared it with a W-220 probe-type sonicator (Heat systems-ultrasonic, Plainview, NY, USA) adjusted to the same output power.

**Figure 1:**
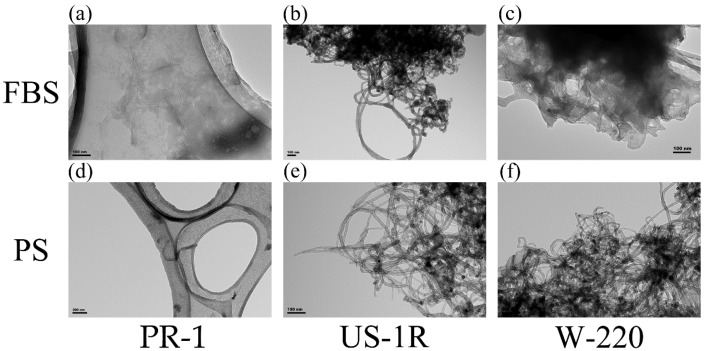
TEM images of Flotube 9110 (FT9110) dispersed by sonication for 1 h. The FT9110 were dispersed in 2% fetal bovine serum (FBS: **a**–**c**), and in 0.1% polysorbate 80 (PS: **d**–**f**) using PR-1 (**a**,**d**), US-1R (**b**,**e**) and W-220 (**c**,**f**) sonicators.

Transmission electron microscope (TEM) images of the FT9110 dispersed using the three types of sonicators in the two dispersants were captured (Figure 1). The PR-1 sonicator dispersed the FT9110 into many single fibers with some tangling (Figure 1a,d) in both dispersants. However, the other two sonicators hardly dispersed the tangled FT9110 into single fibers, regardless of the dispersant (Figure 1b,c,e,f).

The size of the sonicated FT9110 was measured with a Zetasizer Nano ZS (Table 1). FT9110 dispersed in FBS by the PR-1 formed larger clumps than those in PS; the agglomerates were 200 nm and 115 nm in diameter, respectively. Moreover, we confirmed whether FT9110 dispersed by the PR-1 re-agglomerated after time and that FT9110 sonicated with the PR-1 showed signs of physical shear. The size of FT9110 scarcely changed, even after sonication for 5 h or standing for 1 week (Table S1). The size of FT9110 dispersed using the US-1R and W-220 sonicators was on the order of micrometers, and the distribution of the particle size (measured in triplicate), varied greatly and was accompanied by a data quality comment “*X*% particles were out of range” provided by the instrument. FT9110 agglomerates dispersed using the US-1R were larger than those dispersed using the W-220, and PS provided a better dispersal medium than FBS, as found using the PR-1.

### 2.2. Cell Viability

**Figure 2:**
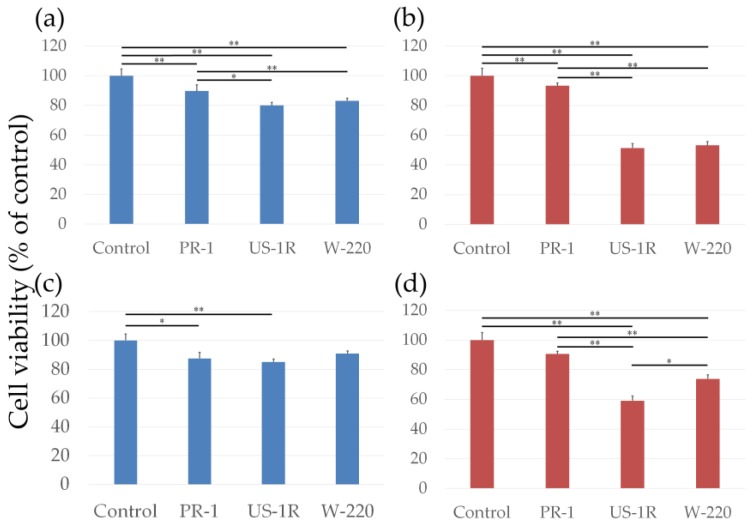
Viability of BEAS-2B cells exposed to 100 μg/mL of Flotube 9110 (FT9110). In (**a**,**b**) the cell viability was measured without FT9110; and in (**c**,**d**) it was measured with FT9110. The FT9110 were dispersed in FBS (**a**,**c**), and in PS (**b**,**d**). Data are expressed as mean ± S.E. (*n* = 6). * *P* < 0.05; ** *P* < 0.01.

First, we measured cytotoxicity using the AlamarBlue assay after removing FT9110 (Figure 2a,b). The viability of cells exposed to FT9110 dispersed in FBS decreased significantly compared to the control, and significant differences were observed between the effects of particles dispersed using the PR-1 and the US-1R or W-220 sonicators (89.7%, 80.0% and 83.1%, respectively). However, for all groups, cell viability was >70%, which is the threshold for non-cytotoxicity as defined by the International Organization for Standardization (ISO) standard. FT9110 dispersed in PS and FBS showed different effects on cell viability. FT9110 dispersed by US-1R and W-220 decreased cell viability to 51.4% and 53.1%, respectively, whereas the effect of FT9110 dispersed using the PR-1 did not vary with the dispersant.

After observing the cells by fluorescence microscopy, we modified the method of the AlamarBlue assay. When cell viability was measured by adding AlamarBlue reagent directly, without excluding the FT9110, FT9110 dispersed in FBS did not affect cell viability, but the viability of cells exposed to FT9110 dispersed in PS by the US-1R and W-220 was increased to 60.3% and 73.3%, respectively (Figure 2c,d).

### 2.3. Observation of Cells by Fluorescence Microscopy

**Figure 3:**
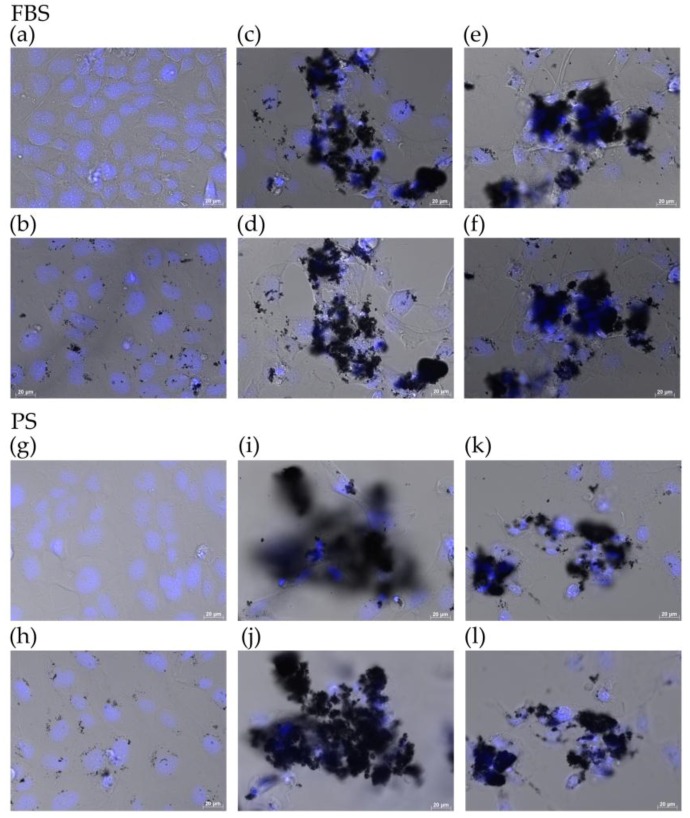
Live cells imaged by differential interference contrast optics after incubation with bisbenzimide H33342 fluorochrome trihydrochloride for nuclear staining in two dispersants. (**a**,**g**) Control; (**b**,**h**) PR-1; (**c**,**i**) US-1R bottom; (**d**,**j**) US-1R top; (**e**,**k**) W-220 bottom; and (**f**,**l**) W-220 top. “Bottom” indicates that the image was captured when the microscope was focused on the bottom of the culture dish. “Top” indicates that the image was captured when the microscope was focused on cells adhered to the top of agglomerated Flotube 9110.

We observed the state of cells exposed to FT9110 with a fluorescent microscope. The cells exposed to FT9110 dispersed using the PR-1, regardless of the dispersant, were adhered to the glass bottom, similar to control cells, and were observed endocytosing FT9110 in the cytoplasm (Figure 3a,b,g,h). Small agglomerates of FT9110, which were not dispersed by sonication, were also observed. Meanwhile, in plates exposed to FT9110 dispersed by the US-1R or the W-220, fewer cells were observed adhered to the glass bottom than in control plates (Figure 3c,e,i,k). Comparing dispersants, in plates exposed to FT9110 in PS, fewer cells were observed adhered to the glass bottom than in plates exposed to FT9110 in FBS, in both the US-1R and W-220 groups. However, some cells were observed adhered to a large agglomerate of FT9110 (Figure 3d,f,j,l).

## 3. Discussion

Sonication and addition of a dispersant are essential to disperse unfunctionalized CNTs, and to evaluate cytotoxicity, because pristine CNTs are hydrophobic and float on water even after vigorous mixing. Moreover, it is well-known that dispersion of tangled MWCNTs is very difficult, even using a proven type of sonicator with high output power. Sonicated CNTs are often not uniformly distributed because supersonic waves show strong directivity and because maximal and minimal points of sound pressure are produced by standing waves. In this study, we tested a new type of sonicator which ameliorates these faults [27]. This sonication technology can maintain the CNT aspect ratio even after mixing, whereas CNTs were broken during conventional Banbury mixing. In addition, we did not observe a reduction in the size of FT9110 agglomerates with time. This effect could clearly be observed in the size of the FT9110 agglomerates, although the FT9110 sonicated by the three different sonicators did not differ in appearance. Figure S1 shows a light microscopy view of FT9110 in culture medium. Moreover, the quantity of FT9110 that we could confirm visually differed substantially between the PR-1 and the US-1R or W-220 groups, because it was difficult to observe the single fibers produced by the PR-1.

We examined two dispersants in this study: FBS and PS. FBS was selected for its similarity to body fluids. In a previous study, researchers sonicated CNTs in culture medium containing FBS directly, and performed the cytotoxicity test by exposing cells to culture medium containing CNTs. However, sonication causes degradation of macromolecular substances, including proteins. Therefore, we sonicated FT9110 in FBS but without culture medium, then added this solution to the culture medium just before use. The other dispersant, PS, was selected because it is typically used as the dispersant for CNTs [28,29]. Our lab has also used PS for in vivo experiments, mainly to avoid provoking an immunoresponse [30,31,32]. Our results showed that PS showed better dispersibility than FBS, using the PR-1. However, because the concentration of the dispersant used was based on our previous experiments using other MWCNTs, and was not optimized for FT9110, the values for FT9110 size are not relevant to further experiments.

Both sonication method and dispersant affected the cytotoxicity of tangled FT9110 individually, and in combination. If we evaluate the cytotoxic effect of the three sonicators using only FBS as the dispersant, the size of the FT9110 agglomerates would lead us to conclude that sonicator type does not affect cytotoxicity. Kim et al. reported that dispersants affect the cytotoxicity of CNTs [33]. We also found that the biological effects of MWCNTs dispersed in the three dispersants differed substantially [19]. Although MWCNTs dispersed with gelatin and DPPC induce significant cytotoxicity, carboxymethyl cellulose (CMC) does not show cytotoxicity in BEAS-2B cells. Moreover, we compared the cytotoxicity of gelatin with FBS as a dispersant for MWNT-7 on normal human bronchial epithelial cells, and FBS showed lower cytotoxicity than gelatin [34]. Liu et al. also reported that single-walled CNT coated with albumin, the most abundant protein in serum, decreased cytotoxicity by reducing cellular uptake [25]. Here, the reason why cytotoxicity was not affected by the size of FT9110 agglomerates dispersed in FBS may be that uptake of CNTs coated with FBS was reduced. In fact, fewer cells adhered to FT9110 in FBS than in PS.

The cytotoxicity of FT9110 dispersed in PS differed significantly between the PR-1 and US-1R or W-220 groups. FT9110 dispersed by the US-1R and W-220 were endocytosed or adhered. Differences in endocytosis and adhesion seem to depend on agglomerate size. It is worthy of note that some of the cells adhered to FT9110 agglomerates may have been alive, because cytotoxicity increased following exclusion of the FT9110 that were adhered to cells, using the AlamarBlue assay. Excluding CNTs in order to avoid absorption of assay by CNTs, which was reported by Casey et al. [35], may cause errors in the evaluation of cytotoxicity under these conditions because the FT9110 concentration was lower and the reaction time was shorter than reported by Casey et al. FT9110 dispersed in PS by the PR-1 did not show cytotoxicity, although slight accumulation of FT9110 was observed in the cells. We have used BEAS-2B cells for evaluation of the cytotoxicity of MWCNTs in many previous experiments [36,37,38,39,40], and in many cases, cytotoxicity occurred via phagocytosis, following excessive intracellular uptake of MWCNTs. In this study, well-dispersed FT9110 did not stimulate phagocytosis. Individual FT9110 are thinner and more flexible than MWNT-7, the standard material for evaluation of the safety of MWCNTs. Interestingly, re-agglomeration was observed after a time, even if the MWNT-7 were dispersed by a PR-1 sonicator (unpublished data), whereas the dispersed state persisted for a long period after the FT9110 were dispersed once. This indicates that tangled MWCNTs differ from agglomerated MWCNTs. Evaluation of the cytotoxicity of tangled MWCNTs, such as FT9110, should take the intended application into account, and the cytotoxicity of tangled MWCNTs should be evaluated separate from that of single fibers.

## 4. Materials and Methods

### 4.1. Suspension and Dispersion of MWCNTs

MWCNT materials were provided by Cnano Technology (FT9110; Santa Clara, CA, USA). The FT9110 were manufactured using a catalytic vapor deposition method; their properties, as provided by the manufacturer, are shown in Table S2 and Figure S2. The FT9110 were sterilized in an autoclave at 121 °C for 15 min and dried, then 10 mg/mL were vortexed in two dispersants (2% FBS (Biowest, Nuaillé, France) in Dulbecco’s phosphate-buffered saline (DPBS) and 0.1% PS (NOF, Tokyo, Japan) in DPBS) [32,41]. Sonication was performed using three different sonicators and their information is shown in Table S3 and Figure S3.

The dispersion state of the FT9110 was observed using a transmission electron microscope (TEM; JEOL, Tokyo, Japan). Sonicated FT9110 were diluted to 1 mg/mL with each dispersant and dipped in a microgrid directly. TEM images were captured at 80 kV.

To determine the hydrodynamic size of the agglomerated FT9110, sonicated FT9110 were measured using a Zetasizer Nano ZS (Malvern Instruments, Worcestershire, UK). The FT9110 were diluted to 1 mg/mL and each measurement was conducted in triplicate.

Dispersed FT9110 at 10 mg/mL were added to cell culture medium at 1/100 volume in each of the following experiments.

### 4.2. Cell Culture

The BEAS-2B human bronchial epithelial cell line was purchased from the American Type Culture Collection (Manassas, VA, USA). BEAS-2B cells were cultured in Ham’s nutrient mixture F-12 (Nacalai Tesque, Kyoto, Japan) with 10% FBS at 37 °C in a 5% CO_2_ humidified incubator and passaged twice per week. For each experiment, the cells were seeded at a density of 3 × 10^5^ cells/mL and allowed to adhere for 24 h.

### 4.3. Cell Viability

Cell viability was assessed using an AlamarBlue assay (alamarBlue^®^ cell viability reagent; Remel, Lenexa, KS, USA). Cells were plated in 96-well plates and incubated for 48 h at 37 °C in culture medium containing 100 μg/mL of FT9110 in a dispersant or in control medium containing only dispersant. After aspiration of the culture medium to exclude the influence of FT9110, 10% AlamarBlue reagent in culture medium was added to each well, where viable cells metabolized the dye for 60 min, resulting in increased fluorescence detected by excitation/emission at 535/590 nm using a plate reader (AF2200; Eppendorf, Hamburg, Germany). Alternatively, AlamarBlue reagent at 10% of the medium volume was simply added to the well without excluding FT9110. Cell viability was calculated as follows: percent cytotoxicity = 100 × experimental value/control value. The media were assayed six times for each treatment condition.

### 4.4. Observation of Cells by Fluorescence Microscopy

Cells cultured on Cellview glass bottom advanced TC 4 compartments (Griner Bio-one, Frickenhausen, Germany) were exposed to FT9110 for 48 h under the same conditions as described for the cell viability assay. For assessment, cells were stained with bisbenzimide H33342 fluorochrome trihydrochloride (H33342, 10 µg/mL; Nacalai Tesque, Kyoto, Japan) for 1 h before observation. The cells were visualized using an AxioObserverZ1 fluorescence microscope (Carl Zeiss Microscopy GmbH, Jena, Germany) with a 40× objective lens.

### 4.5. Statistical Analysis

Data are presented as mean ± S.E. Statistical significance was determined by analysis of variance followed by the Tukey-Kramer method for comparisons between different types of sonicators. *P*-value < 0.05 was considered statistically significant.

## 5. Conclusions

We separated tangled FT9110 into single fibers and evaluated their cytotoxicity. Well-dispersed FT9110 showed almost no cytotoxicity. The cytotoxicity of FT9110 was found to differ with the degree of agglomeration following sonication, and to depend on the dispersant. In conclusion, we suggest that the cytotoxicity of hydrophobic nanomaterials, such as carbon nanomaterials, be evaluated under the optimum conditions, based on the intended application of each nanomaterial.

## Figures and Tables

**Table 1:** The size of Flotube 9110 agglomerates measured by Zetasizer.

Sonicator	Z-Average (d, nm)	
	Fetal Bovine Serum	Polysorbate 80
PR-1	200 ± 27	116 ± 0
US-1R	4434 ± 341	5100 ± 488
W-220	1603 ± 113	2781 ± 512

## Supplementary Materials

The following are available online at http://www.mdpi.com/2079-4991/6/11/219/s1.

## Abbreviations

CNT	Carbon nanotube
MWCNT	Multi-walled carbon nanotube
FT9110	Flotube 9110
BSA	Bovine serum albumin
PS	Polysorbate 80
DPPC	Dipalmitoylphosphatidylcholine
FBS	Fetal bovine serum
DPBS	Dulbecco’s phosphate-buffered saline
TEM	Transmission electron microscope
H33342	Bisbenzimide H33342 fluorochrome trihydrochloride

## References

[1] Behabtu N., Young C.C., Tsentalovich D.E., Kleinerman O., Wang X., Ma A.W., Bengio E.A., ter Waarbeek R.F., de Jong J.J., Hoogerwerf R.E. (2013). Strong, light, multifunctional fibers of carbon nanotubes with ultrahigh conductivity. Science.

[2] Cao Q., Han S.J., Tulevski G.S., Zhu Y., Lu D.D., Haensch W. (2013). Arrays of single-walled carbon nanotubes with full surface coverage for high-performance electronics. Nat. Nanotechnol..

[3] Ganzhorn M., Klyatskaya S., Ruben M., Wernsdorfer W. (2013). Strong spin-phonon coupling between a single-molecule magnet and a carbon nanotube nanoelectromechanical system. Nat. Nanotechnol..

[4] De Volder M.F., Tawfick S.H., Baughman R.H., Hart A.J. (2013). Carbon nanotubes: Present and future commercial applications. Science.

[5] Saito N., Haniu H., Usui Y., Aoki K., Hara K., Takanashi S., Shimizu M., Narita N., Okamoto M., Kobayashi S. (2014). Safe clinical use of carbon nanotubes as innovative biomaterials. Chem. Rev..

[6] Saito N., Usui Y., Aoki K., Narita N., Shimizu M., Hara K., Ogiwara N., Nakamura K., Ishigaki N., Kato H. (2009). Carbon nanotubes: Biomaterial applications. Chem. Soc. Rev..

[7] Mehra N.K., Mishra V., Jain N.K. (2014). A review of ligand tethered surface engineered carbon nanotubes. Biomaterials.

[8] Zarschler K., Rocks L., Licciardello N., Boselli L., Polo E., Garcia K.P., De Cola L., Stephan H., Dawson K.A. (2016). Ultrasmall inorganic nanoparticles: State-of-the-art and perspectives for biomedical applications. Nanomedicine.

[9] Bakand S., Hayes A. (2016). Toxicological considerations, toxicity assessment, and risk management of inhaled nanoparticles. Int. J. Mol. Sci..

[10] Bouwmeester H., Hollman P.C., Peters R.J. (2015). Potential health impact of environmentally released micro- and nanoplastics in the human food production chain: Experiences from nanotoxicology. Environ. Sci. Technol..

[11] Takagi A., Hirose A., Nishimura T., Fukumori N., Ogata A., Ohashi N., Kitajima S., Kanno J. (2008). Induction of mesothelioma in p53+/− mouse by intraperitoneal application of multi-wall carbon nanotube. J. Toxicol. Sci..

[12] Takagi A., Hirose A., Futakuchi M., Tsuda H., Kanno J. (2012). Dose-dependent mesothelioma induction by intraperitoneal administration of multi-wall carbon nanotubes in p53 heterozygous mice. Cancer Sci..

[13] Nagai H., Okazaki Y., Chew S.H., Misawa N., Yamashita Y., Akatsuka S., Ishihara T., Yamashita K., Yoshikawa Y., Yasui H. (2011). Diameter and rigidity of multiwalled carbon nanotubes are critical factors in mesothelial injury and carcinogenesis. Proc. Natl. Acad. Sci. USA.

[14] Suzui M., Futakuchi M., Fukamachi K., Numano T., Abdelgied M., Takahashi S., Ohnishi M., Omori T., Tsuruoka S., Hirose A. (2016). Multiwalled carbon nanotubes intratracheally instilled into the rat lung induce development of pleural malignant mesothelioma and lung tumors. Cancer Sci..

[15] Nagai H., Okazaki Y., Chew S.H., Misawa N., Miyata Y., Shinohara H., Toyokuni S. (2013). Intraperitoneal administration of tangled multiwalled carbon nanotubes of 15 nm in diameter does not induce mesothelial carcinogenesis in rats. Pathol. Int..

[16] Wirnitzer U., Herbold B., Voetz M., Ragot J. (2009). Studies on the in vitro genotoxicity of baytubes, agglomerates of engineered multi-walled carbon-nanotubes (MWCNT). Toxicol. Lett..

[17] Catalán J., Siivola K.M., Nymark P., Lindberg H., Suhonen S., Järventaus H., Koivisto A.J., Moreno C., Vanhala E., Wolff H. (2016). In vitro and in vivo genotoxic effects of straight versus tangled multi-walled carbon nanotubes. Nanotoxicology.

[18] Haniu H., Saito N., Matsuda Y., Tsukahara T., Usui Y., Maruyama K., Takanashi S., Aoki K., Kobayashi S., Nomura H. (2014). Biological responses according to the shape and size of carbon nanotubes in BEAS-2B and MESO-1 cells. Int. J. Nanomed..

[19] Haniu H., Saito N., Matsuda Y., Kim Y.A., Park K.C., Tsukahara T., Usui Y., Aoki K., Shimizu M., Ogihara N. (2011). Effect of dispersants of multi-walled carbon nanotubes on cellular uptake and biological responses. Int. J. Nanomed..

[20] Nel A.E., Mädler L., Velegol D., Xia T., Hoek E.M., Somasundaran P., Klaessig F., Castranova V., Thompson M. (2009). Understanding biophysicochemical interactions at the nano-bio interface. Nat. Mater..

[21] Cedervall T., Lynch I., Lindman S., Berggård T., Thulin E., Nilsson H., Dawson K.A., Linse S. (2007). Understanding the nanoparticle-protein corona using methods to quantify exchange rates and affinities of proteins for nanoparticles. Proc. Natl. Acad. Sci. USA.

[22] Mahmoudi M., Lynch I., Ejtehadi M.R., Monopoli M.P., Bombelli F.B., Laurent S. (2011). Protein-nanoparticle interactions: Opportunities and challenges. Chem. Rev..

[23] Ge C., Du J., Zhao L., Wang L., Liu Y., Li D., Yang Y., Zhou R., Zhao Y., Chai Z. (2011). Binding of blood proteins to carbon nanotubes reduces cytotoxicity. Proc. Natl. Acad. Sci. USA.

[24] El-Sayed R., Bhattacharya K., Gu Z., Yang Z., Weber J.K., Li H., Leifer K., Zhao Y., Toprak M.S., Zhou R. (2016). Single-walled carbon nanotubes inhibit the cytochrome p450 enzyme, CYP3A4. Sci. Rep..

[25] Liu Y., Ren L., Yan D., Zhong W. (2014). Mechanistic study on the reduction of SWCNT-induced cytotoxicity by albumin coating. Part. Part. Syst. Charact..

[26] Shannahan J.H., Brown J.M., Chen R., Ke P.C., Lai X., Mitra S., Witzmann F.A. (2013). Comparison of nanotube-protein corona composition in cell culture media. Small.

[27] Tsuchiya K., Sakai A., Nagaoka T., Uchida K., Furukawa T., Yajima H. (2011). High electrical performance of carbon nanotubes/rubber composites with low percolation threshold prepared with a rotation–revolution mixing technique. Compos. Sci. Technol..

[28] Muller J., Huaux F., Moreau N., Misson P., Heilier J.F., Delos M., Arras M., Fonseca A., Nagy J.B., Lison D. (2005). Respiratory toxicity of multi-wall carbon nanotubes. Toxicol. Appl. Pharmacol..

[29] Patlolla A., Patlolla B., Tchounwou P. (2010). Evaluation of cell viability, dna damage, and cell death in normal human dermal fibroblast cells induced by functionalized multiwalled carbon nanotube. Mol. Cell. Biochem..

[30] Takanashi S., Hara K., Aoki K., Usui Y., Shimizu M., Haniu H., Ogihara N., Ishigaki N., Nakamura K., Okamoto M. (2012). Carcinogenicity evaluation for the application of carbon nanotubes as biomaterials in rash2 mice. Sci. Rep..

[31] Nomura H., Takanashi S., Tanaka M., Haniu H., Aoki K., Okamoto M., Kobayashi S., Takizawa T., Usui Y., Oishi A. (2015). Specific biological responses of the synovial membrane to carbon nanotubes. Sci. Rep..

[32] Shimizu M., Kobayashi Y., Mizoguchi T., Nakamura H., Kawahara I., Narita N., Usui Y., Aoki K., Hara K., Haniu H. (2012). Carbon nanotubes induce bone calcification by bidirectional interaction with osteoblasts. Adv. Mater..

[33] Kim J.S., Song K.S., Lee J.H., Yu I.J. (2011). Evaluation of biocompatible dispersants for carbon nanotube toxicity tests. Arch. Toxicol..

[34] Maruyama K., Haniu H., Saito N., Matsuda Y., Tsukahara T., Kobayashi S., Tanaka M., Aoki K., Takanashi S., Okamoto M. (2015). Endocytosis of multiwalled carbon nanotubes in bronchial epithelial and mesothelial cells. Biomed. Res. Int..

[35] Casey A., Herzog E., Davoren M., Lyng F.M., Byrne H.J., Chambers G. (2007). Spectroscopic analysis confirms the interactions between single walled carbon nanotubes and various dyes commonly used to assess cytotoxicity. Carbon.

[36] Haniu H., Saito N., Matsuda Y., Kim Y.A., Park K.C., Tsukahara T., Usui Y., Aoki K., Shimizu M., Ogihara N. (2011). Elucidation mechanism of different biological responses to multi-walled carbon nanotubes using four cell lines. Int. J. Nanomed..

[37] Haniu H., Saito N., Matsuda Y., Usui Y., Aoki K., Shimizu M., Ogihara N., Hara K., Takanashi S., Okamoto M. (2012). Manufacturing strategy for multiwalled carbon nanotubes as a biocompatible and innovative material. J. Nanotechnol..

[38] Tsukahara T., Haniu H. (2011). Cellular cytotoxic response induced by highly purified multi-wall carbon nanotube in human lung cells. Mol. Cell. Biochem..

[39] Tsukahara T., Matsuda Y., Usui Y., Haniu H. (2013). Highly purified, multi-wall carbon nanotubes induce light-chain 3b expression in human lung cells. Biochem. Biophys. Res. Commun..

[40] Haniu H., Saito N., Matsuda Y., Tsukahara T., Maruyama K., Usui Y., Aoki K., Takanashi S., Kobayashi S., Nomura H. (2013). Culture medium type affects endocytosis of multi-walled carbon nanotubes in beas-2b cells and subsequent biological response. Toxicol. In Vitro.

[41] Jacobsen N.R., Pojano G., Wallin H., Jensen K.A. (2010). Nanomaterial Dispersion Protocol for Toxicological Studies in Enpra.

