# Supplementary material for: The Dispersion State of Tangled Multi-Walled Carbon Nanotubes Affects Their Cytotoxicity
Source: Nanomaterials (Basel). 2016 Nov 19;6(11):219. doi: 10.3390/nano6110219 (PMC5245756; doi:10.3390/nano6110219)
Supplement: Supplementary file 1 [file nanomaterials-06-00219-s001.pdf]

Supplementary Materials: The Dispersion State of Tangled Multi-Walled Carbon Nanotubes Affects Their Cytotoxicity

Chika Kuroda, Hisao Haniu, Kumiko Ajima, Manabu Tanaka, Atsushi Sobajima, Haruka Ishida, Tamotsu Tsukahara, Yoshikazu Matsuda, Kaoru Aoki, Hiroyuki Kato and Naoto Saito

Table S1. The size of FT9110 sonicated by PR-1.

	**Z-average (d, nm)**	
	**FBS**	**PS**
5 h	290 ± 2	104 ± 0
1 week	210 ± 4	122 ± 1

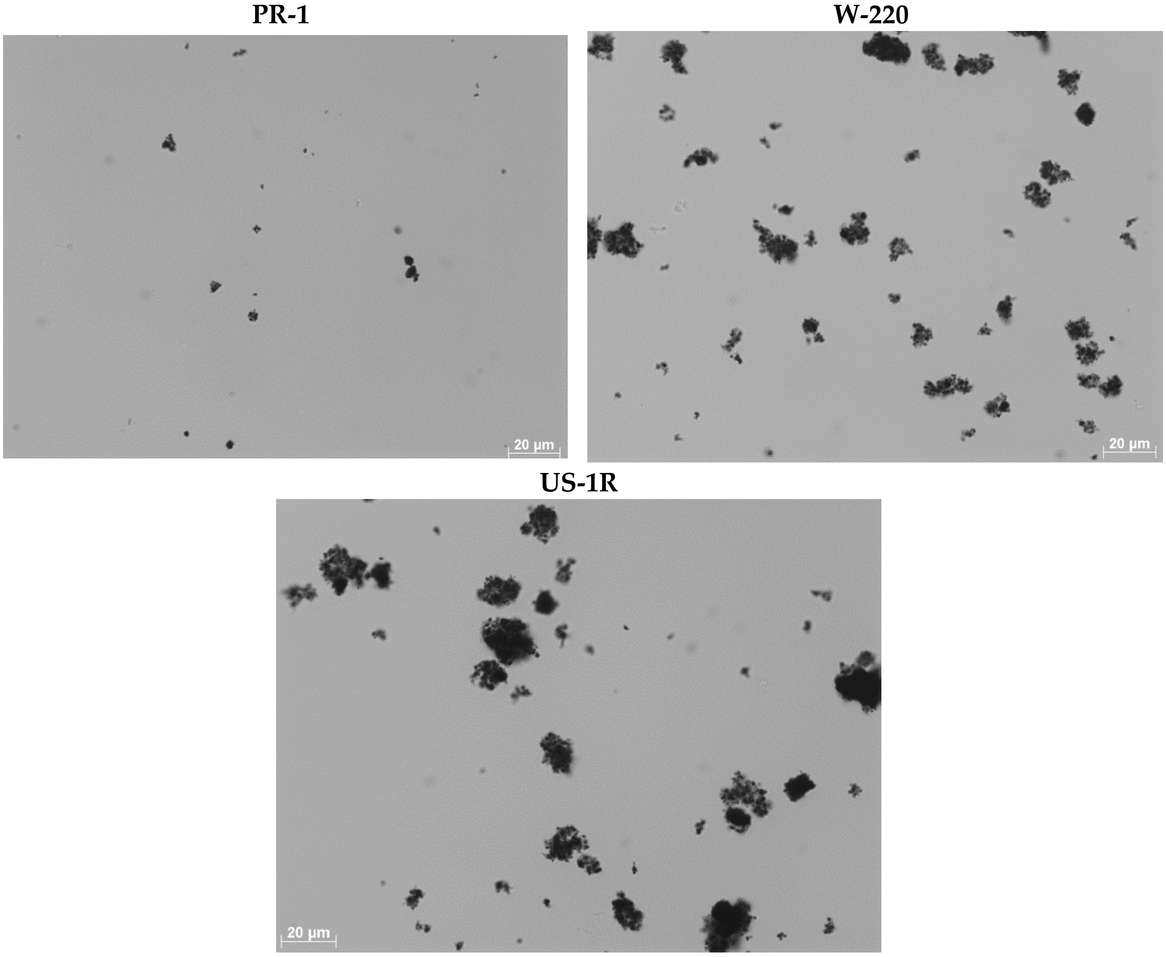

Figure S1. Light microscopic view of dispersed FT9110:FT9110 dispersed in PS by each sonicator were added to culture medium at 1/100 volume on Cell view glass bottom advanced TC 4 compartments. After 1 h, a light microscopic view was obtained using an AxioObserverZ1 fluorescence microscope.

Table S2. Properties of FT 9110.

Properties	Units	Values
Average diameter	nm	10–15
Length	mm	10
Purity	%	≥99.8
Non-carbon content	%	≤0.2
Fe elemental content	ppm	>100
Surface area (BET)	M2/g	160–230
Tap density	g/cm2	0.1–0.2

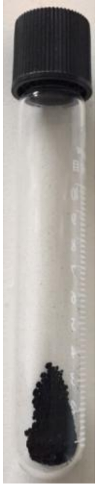

Figure S2. FT 9110.

Table S3. Sonicator information.

Property	PR-1	US-1R	W-220
Picture	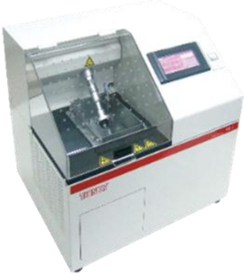	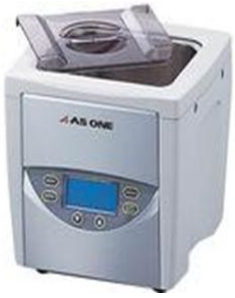	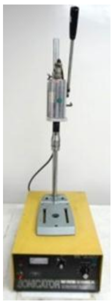
Manufacturer	Thinky (Tokyo, Japan)	As one (Tokyo, Japan)	Heat systems-ultrasonic (Plainwiew, NY, USA)
Output power	140 W	55 W	140 W
Sonicator type	Water bath	Water bath	Prove

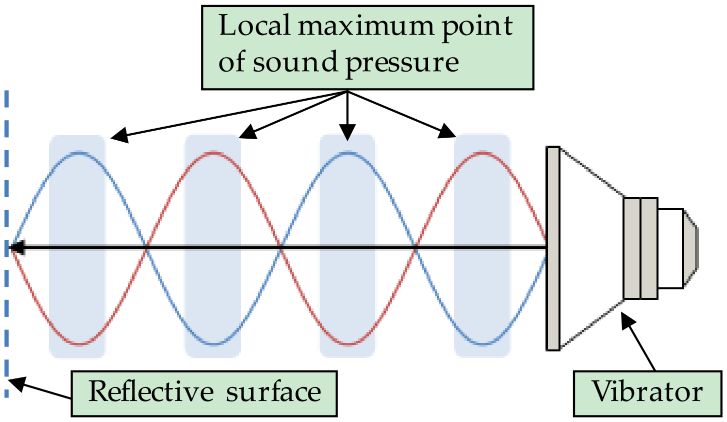

(**A**)

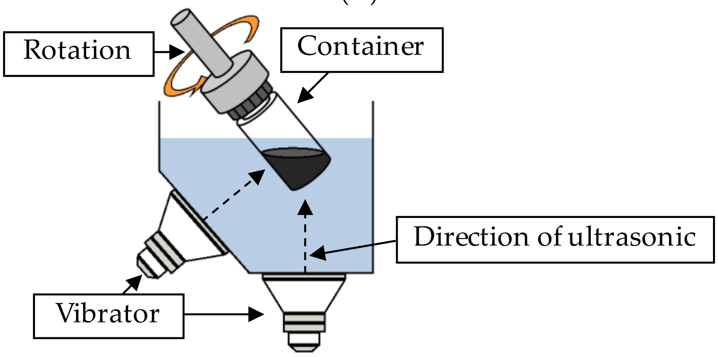

(**B**)

Figure S3. PR-1 Nano Premixer information**:** PR-1 is an ultrasonic sonicator that can stir CNTs and buffer by rotating the container. This structure enables uniform ultrasonic irradiation of samples and suppresses the uneven dispersion caused by standing waves (**A**); the two vibrators located on the bottom and the side enhance dispersion efficiency by concentrating the ultrasonic radiation (**B**). The PR-1 has more than double the ultrasonic output power of conventional sonicators, even though it is smaller than conventional sonicators; it is also easy to use because it uses an ultrasonic bath system. This mechanism and structure increase the watt density and improve the dispersion performance.

© 2016 by the authors. Submitted for possible open access publication under the terms and conditions of the Creative Commons Attribution (CC-BY) license (http://creativecommons.org/licenses/by/4.0/).
